# Allergy to natural rubber latex in asthma patients

**DOI:** 10.1186/2045-7022-1-S1-P65

**Published:** 2011-08-12

**Authors:** Kseniya Uspenskaia, Ludmila Luss, Alexander Babakhin

**Affiliations:** 1 NRC Institute of immunology FMBA Russia, out- patient, Moscow, Russian Federation; 2 RNC Intitute of Immunology FMBA Russia, Out-patient, Moscow, Russian Federation; 3 RNC Intitute of Immunology FMBA Russia, Moscow, Russian Federation

## 

Natural rubber latex (NRL) allergy has become an important occupational health problem in recent years. However, the prevalence of sensitization to NRL among allergic asthmatic patients is unknown. This study was undertaken evaluate the levels specific IgE in sera of 70 adult asthmatic patients (27 male and 43 female) with mild (32 patients) and moderate (38 patients) asthma, whose diagnosis was confirmed by case history, skin test results and RAST 20% of the asthmatics (14 patients) had latex-specific IgE in sera detected by Pharmacia UniCAP. Among the 14 latex sensitized asthmatics 6 patients had mild asthma and 8 had moderate asthma, 10 being male and 4 female. No latex-specific IgE was found in sera of 10 healthy donors. Among the 14 latex-sensitized asthmatics, 12 (86%) had sensitization to house dust; 13 (93%) sensitization to trees; 11 (79%) sensitization to grasses; 11 (79%) sensitization to weeds; 4 (29%) sensitization to cat/dog. There are no correlation between sensitivity to latex determined by latex specific IgE in sera and occupation of these asthma patients. 12 patients among the latex-sensitized asthmatics (86%0 had banana-specific IgE. 50 (72%) of the asthma patients had high levels of total IgE and all latex-sensitized asthmatics had high levels of total IgE. Using a one point RAST inhibition assay employing 10mg/ml of Greer latex extract 50% RAST inhibition was obtained, confirming specificity of the anti-latex IgE in these asthmatics. This study demonstrated a relatively high prevalence (20%) of in vitro reactivity to NRL allergens among patients with asthma. The source of sensitization to latex in these patients remains to be determined.

